# Cosmopolitan enclaves: An introduction

**DOI:** 10.1177/0308275X211059659

**Published:** 2021-11-06

**Authors:** Jeanne Rey, Matthieu Bolay, Yonatan N Gez

**Affiliations:** 129273 University of Teacher Education, Fribourg, Switzerland; 30525 The Graduate Institute of International and Development Studies, Geneva, Switzerland; Arnold Bergstraesser Institute, Freiburg-im-Breisgau, Germany; 30525 The Graduate Institute of International and Development Studies, Geneva, Switzerland

**Keywords:** Boundary work, cosmopolitanism, education, enclave, expatriates, globalization, migration, mobility

## Abstract

Cosmopolitan enclaves emerge at the intersection of global dynamics and local contexts as spaces where the cultivation of a cosmopolitan ethos encounters processes of socio-spatial boundary work and segregation. In the introduction to this special issue, we discuss under which circumstances the intention to cultivate open-mindedness goes hand in hand with keeping the local environment at bay. We argue that ethnographic attention to cosmopolitan enclaves may help bridge macro-level observations regarding globalization and its graduated sovereignties with the micro-level understanding of actual day-to-day interactions and boundary work within concrete spaces. We thus address the paradox of the omnipresence of enclaves in a global world and analyse the ambiguous aspirations and expectations derived from cosmopolitan ideals and how they relate to (under)privilege. While cosmopolitan aspirations exist alongside reproductions of postcolonial representations and hierarchies, they may also express the will to resist the politics of exclusion by demarcating an alternative safe haven.

This special issue explores the paradoxical intersection between the cultivation of a cosmopolitan ethos and processes of socio-spatial boundary work and segregation.^1^ That the two often intersect within a single project or space leads us to introduce the concept of cosmopolitan enclaves, where the cultivation of open-mindedness goes hand in hand with keeping the local environment at bay (Rey et al., 2019; Wagner, 1998; Weenink, 2008). As this volume’s ethnographic case studies show, a focus on the spatial grounding of the political and economic projects where cosmopolitan claims are concretized highlights counterintuitive processes of closure and socio-spatial segregation. By scrutinizing empirical contexts where both cosmopolitanism and enclavement take place, ethnographic accounts can bridge macro-level observations regarding globalization and its graduated sovereignties^2^ with the micro-level understanding of day-to-day interactions and boundary work within concrete spaces. From the uneven economic structures within which cosmopolitan ideals unfold to the educational, professional, legal, and political spaces in which they are enacted in practice, this perspective acknowledges that the ambiguous aspirations and expectations derived from cosmopolitan ideals are shared across a wide social spectrum and are neither a prerogative of so-called transnational elites nor a subjugated condition of subaltern strata. By empirically enlarging the focus to a wide range of enclave spaces, the contributions in this issue draw attention to how cosmopolitanism may partake both in the reproduction and contestation of privilege, power, and social hierarchies of class and race.

The notion of enclave comes from geography, where it is used to designate ‘the existence of a fragment enclosed in something of an alien nature’ (Vinokurov, 2007: 9). A common example within political geography would be a territory within one country that is administered by another, while an example from economic geography would be a foreign-dominated industry within a local national economy. A more dynamic use of the notion surged in sociological and anthropological analysis of globalization, where it is used to account for the global fragmentation of space, the proliferation of differentiated sovereignty, and the pivotal role of exception spaces in the context of global flows of people and capital. Works on specific enclave economies – such as in the sectors of commerce and extraction (Bräutigam and Xiaoyang, 2012; Ferguson, 2005) – render especially salient how processes of enclavement go hand in hand with the expansion of transnational supply chains whose networks span across financial centres and capitalist frontiers (Chalfin, 2010; Watts, 2019). While enclaves foster the circulation of people, goods, and capital, they do so by offering a certain degree of ‘immunity’ (Donner, 2011) from local/national administrative – and legal – control and accountability.

The revival of scholarly interest in enclaves is also found in the field of migration studies, where the concept of ‘ethnic enclaves’ (Portes and Manning, 1986) gained momentum in the 1990s in the context of debates about assimilation theory. The ethnic enclave hypothesis posited that subaltern immigrant groups used segregation based on ethnic ties to develop independent economies that circumvented labour market discriminations, and in so doing, actually resulted in better ‘assimilation’. As Waldinger (1993: 448) observed, the ethnic enclave hypothesis turns ‘the concept of the enclave economy upside down’. Rather than the cores establishing spaces of production and circulation in the peripheries that have virtually no ties to the domestic economy, peripheries would subvert and reverse this logic by establishing bottom-up, ethnically bounded spaces of production and circulation within the cores. Such urban patchworks would result not only in ‘new patterns of segregation’ (Vertovec, 2007: 1046) but also in ‘new forms of cosmopolitanism [marked by] multiple cultural competences […] and practices of code switching’ (Vertovec, 2007: 1046).

Similarly to such prior conceptualizations of enclaves, cosmopolitan enclaves are characterized by dual dynamics of bordering and circulation. They facilitate the global circulation of specific actors across enclaves, while maintaining (relative) detachment from local social, political and legal conditions and constraints. However, in contrast to conceptions of enclave economies, where strategies of relative socio-spatial closure tend to be explicitly acknowledged (e.g. Gilbert, 2018), cosmopolitan enclaves are marked by discourses of openness, ideals of universality, and imaginaries of an integrated and inclusive world. These ideals and values may appear universal while actually drawing on specific cultural forms (notably Western and capitalist), with de facto adverse consequences for inclusion. More clearly than in the case of ‘ethnic enclaves’, ‘cosmopolitan enclaves’ take the form of decentralized and dynamic networks, where actors can opt in and out, or enact ambiguous belonging.

The concept of cosmopolitan enclave helps to address the link between enclavement, cultural performance, and (under)privilege. The articles included in this special issue focus on social, economic and legal processes of construction of enclave boundaries at the local and national levels. They analyse the stances that develop inside enclaves towards the immediate and supposedly non-cosmopolitan environment, and tease out the practices of inclusion and exclusion by which social and spatial boundaries are constructed. The articles also address the (de)localization of cosmopolitan social spaces and the resources and capitals accrued through the mobilization of cosmopolitan ideals. Questioning the equation between cosmopolitan enclaves and (under)privilege, the case studies presented in this special issue range from high-end expatriate communities to international schools and multinational companies. Conversely, the authors also consider how cosmopolitan enclavement may be strategically constructed or feigned within mixed underprivileged neighbourhoods or marginalized townships as a means to access resources through (the performance of) greater connectedness.

## Cosmopolitanism as a cultural hallmark of globalization

As anthropologists have noted over the last decades, rapid economic, social, technological and political transformations have dramatically changed life-worlds and reshaped space and territoriality. These developments challenged the sovereignty of the state and other political structures and their established boundaries. The drivers of these changes – shorthanded as globalization – include rapid transformations in technology, politics of movement (of capital, goods, information or population) or global discourses and regulations (Wydra and Thomassen, 2018).

Cosmopolitan enclaves represent both an epiphenomenon and an index of globalization. The territories that they create tend to be constructed around cultural, linguistic, and axiological practices and set to transcend national and local systems and rules. Going further than the critique of cosmopolitanism as an elite practice, the articles in this volume show that cosmopolitan enclaves emerge in relation to a variety of local particularities: the aspirations for a better life by those who feel abandoned by a mystifying globalization; the political activism of citizens aiming to defeat the ambient xenophobic climate; the domestic and infrastructural needs of the privileged segment of the working elite of globalization. Thus, cosmopolitan stances tend to cultivate a meta-narrative on identity politics that opposes the seeming salience of identity withdrawal in the wake of the renewed appeal of populisms and nationalisms around the globe (Agier, 2013; Ward, 2020). By contrast, within cosmopolitan enclaves, nation states may be looked down upon as relics of the past, as a potential source of dangerous political extremism, or as territories to be crossed, explored, exploited or reformed.

### Cosmopolitanism and cultural critique

Anthropologists have developed an ambivalent attitude towards cosmopolitanism, at times praising it as a political and moral horizon promoting coexistence and at times suspecting it of hoodwinking and distracting from growing inequalities. While these views are not necessarily contradictory, they tend to refer to distinct starting points and narratives. Cosmopolitanism opposes radical identity politics such as nationalism or racial supremacism, to which it offers a moral response and a political alternative. The capacity to relate to a plurality of cultures (Hannerz, 1990) as well as the tendency within post-identity politics to overlap between ‘interests and heterogenous or hybrid publics in order to challenge conventional notions of belonging, identity and citizenship’ (Vertovec and Cohen, 2002: 1) represent a refreshing and timely political horizon, sometimes inspired by a postcolonial perspective (Bhabha, 1993). In parallel to the moral call for transcending identity politics by recognizing shared interdependencies within a single world-community (e.g. Appiah, 2006) and ‘commit[ting] to a common humanity’ (Glick Schiller, 2015: 32), cosmopolitanism also implies a practical critique of ‘methodological nationalism’ in the social sciences. Against methodological nationalism, cosmopolitanism sets a ‘methodological cosmopolitanism’ that would overcome global/local, national/international, or us/them binaries (Beck and Sznaider, 2010: 382). And yet, cosmopolitanism is not ‘a view from nowhere’ as critics have argued: while genealogies of cosmopolitanism can be traced back to a great variety of historical and social contexts (Tihanov, 2015), dominant views remain tied to a Eurocentric ‘ontology of Kantian cosmopolitanism’ (Uimonen, 2020: 83), that is, an implicitly evolutionist worldview of a common humanity whose model should be the European liberal state.

The emergence of cosmopolitanism is situated within a long history of colonialism nurtured by a European cultural elite (Van der Veer, 2002). Today, still, colonial legacies and imperialist attitudes might haunt the privileged hypermobile transnationals (Yeoh and Willis, 2005) and cosmopolitan stances be touched by latent colonial baggage. From this perspective, cosmopolitanism may well be instrumental in reproducing power relations and inequalities along with the expansion of neoliberal regimes. Cosmopolitan attitudes have thus also become the new hallmark of an emerging social class that constitutes the executive workforce of economic globalization (Bolay and Rey, 2020; Calhoun, 2002; Ye and Kelly, 2011). While these hypermobile transnationals discursively frame their social relations as open to the ‘cultural Other’, they tend to adopt a certain blindness towards their own class habitus, which de facto excludes as much as it includes.

### Cosmopolitanism and cultural capital

From this latter perspective, education represents a good entry point for analysing the articulation between cosmopolitanism, globalization and social stratification. Igarashi and Saito (2014) state that educational institutions help to institutionalize cosmopolitanism as a form of cultural capital signalling embodied dispositions to extensively interact with people of multiple nationalities and cultures. These dispositions are required to access privileged positions in global arenas. In this sense, cosmopolitan attitudes contribute to the process of social stratification. They tend to amplify or consolidate inequalities by constructing a specific form of cultural capital, whose attributes are unequally distributed within a population (Igarashi and Saito, 2014: 223). Thus, in educational contexts, cosmopolitanism, seen as a marker of distinction, participates in the ‘subtle and not so subtle ways that formally meritocratic institutions help to recreate systems of social stratification’ (Lamont and Lareau, 1988: 155; see also Friedman, 2018). As part of their habitus, cosmopolitan attitudes cultivated in international schools or by education abroad contribute to the sense of group belonging to a transnational elite composed not only of expatriates, but also of cosmopolitan locals who have acquired the marks of internationality (Rey et al., 2019; Waters, 2007; Weenink, 2008).

Beyond privilege, anthropologists note that ‘cosmopolitanism from below’ represents another facet of cosmopolitanism. From the ‘ordinary cosmopolitanism’ of working-class men (Lamont and Aksartova, 2002) through Senegalese Mouride traders’ ‘vernacular cosmopolitanism’ (Diouf, 2000) to ‘cosmopolitanism from below’ among activists of the alternative globalization movement (Kurasawa, 2004), bottom-up cosmopolitanism is often depicted as ethical or political rather than distinctive or instrumental. Thus, there tends to be a gap in the conceptualization of cosmopolitanism according to class position. As Werbner asks: ‘are we talking about non-elite [forms of cosmopolitanism], or of non-European but nevertheless high cultures produced and consumed by non-western elites?’ (2006: 497).

Thus, among elites, cosmopolitanism is often framed as a resource or a form of capital that can support individual or class strategies. Among grassroots movements, cosmopolitanism is, by contrast, referred to as facilitating the expansion of horizons, borne out of exclusion rather than opportunity (Appadurai, 2013), yet is rarely framed as a resource. From a critical field theory, one reason for that might lie in the greater difficulty of converting cosmopolitanism from below into other forms of capital. For instance, Wagner (2020) notes that cosmopolitan attitudes and experiences may be recognized as a form of cultural capital for privileged social groups, while the same attitudes and experiences may be stigmatized among lower social classes. Thus, cosmopolitan attitudes as a form of cultural capital can only work as a multiplier of previously existing economic, cultural and social capitals. Alternatively, Werbner (2006) suggests that cosmopolitanism from below may well assert distinction among non-elites or non-European groups, but is only recognized as such within the boundaries of these groups. In this regard, Cacciotti’s article in this issue presents an interesting case where she argues that the distinctive nature of cosmopolitanism does not lead to a stronger social stratification but rather to an extended sense of belonging (in her case, beyond the Italian/foreigner dichotomy). Her work shows how ostracization and exclusion is contested, and how political mobilization relies on migrant populations’ potential to be considered through the lenses of diversity and cosmopolitanism and thereby become a resource.

## Enclaves for a global world: Exploring the paradox

By scrutinizing cosmopolitanism’s dynamics of inclusion/exclusion in contexts of relative social and spatial boundedness, we wish to attend to the inherent tension between claims of (socio-cultural) openness and practices of (spatial) closure. This points to the ambivalences and contradictions that are found in the various case studies presented in this special issue. Whoever the subject of inquiry is – expatriates in Dubai, southern China, Maputo, or underprivileged families in the slums of Nairobi or the marginalized neighbourhoods of Rome – cosmopolitan aspirations exist alongside missed encounters, cultural misunderstandings, disillusions and reproductions of postcolonial representations and status hierarchies. They may also express the will to resist the politics of exclusion and alienation of minorities and foreigners by demarcating an alternative safe haven.

### Enclaves and boundary work

Underlying the notion of cosmopolitan enclaves is the idea that social and cultural boundaries are articulated in relation to specific spaces. This spatial dimension of boundaries related to cosmopolitanism requires further attention: Donnan and Wilson (1994) indeed note that while geopolitical territorial boundaries are always also cultural and symbolic, cultural and symbolic boundaries do not necessarily have a spatial dimension. Yet, this spatial dimension of social and symbolic boundaries comes to the forefront in this special issue. Enclaves differ from the surrounding space through differentiated access and by the nature or the intensity of economic activities, the language spoken, the formal or informal dominant institutions, and the rules that govern them. These differences are often materialized by visual and/or architectural elements that define an enclave’s physical limits. In this regard, Ballif and Rosière (2009) analyse the politics of walling (*teichnopolitics*), which paradoxically proliferate in the era of globalization. *Teichnopolitics* deals with concerns about security, but also with the defence of economic interests. Be it by walls, as in gated communities, or by ‘softer’ social and symbolic markers, as in immigrant neighbourhoods, many cities in the 21st century developed into what Murray terms an assemblage of enclaves:

For the privileged, tapping into new sources of wealth and power has enabled them to insulate themselves in virtually sealed enclaves disconnected from the material deprivations of the everyday urbanism that surrounds them. […] The retreat of the wealthy and propertied behind walls and gates has gone hand-in-glove with the relegation of the poor into spaces of confinement at the margins of the mainstream urban life. (Murray, 2017: 99)

Likewise, the pervasiveness of enclavement processes at both extremities of the class spectrum is also salient in the contributions to this issue. Despite profound differences regarding the social and cultural composition of the population that inhabit them, these cosmopolitan enclaves share articulations of socio-spatial segregation – including both material bordering and symbolic distinction – combined with ideologies of openness and boundary-crossing.

This is most obvious in the cases of privileged expatriate communities, such as in the articles written by Assa-Inbar on international education in China, by Botelho on work relations between expatriates and local household workers in Mozambique, and by Cosquer on how expatriates’ aspirations for cosmopolitan sociality in Abu Dhabi are framed and de facto limited by orientalist conceptions. By scrutinizing expatriates’ segregated lives beyond their urban and material boundaries, or the class- and race-based homophily that characterizes their networks, these contributions shed light on the ambiguous aspirations of expatriates to transcend these boundaries, even as the ethical negotiations in which they engage ultimately seem to reinforce them. In such contexts, where material boundaries remain prominent, contact with the surrounding territory is presented as functionally superfluous but ethically desirable.

Yet, to allow people to spend their life secluded in an enclaved space – be it a gated community, a mining compound, or an immigrant neighbourhood – requires a constant work of construction, internal regulation, and maintenance of boundaries. It also requires an intense mobility across the enclave boundaries to provide its inhabitants with the necessary infrastructure, goods, and services. In privileged cosmopolitan enclaves, this tends to be provided by ‘the locals’ who are employed as household workers, drivers, or guards. This dynamic is examined in Botelho’s article, in which relations between Scandinavian expats and local domestic workers in Maputo become a vivid illustration of expatriates’ failure to impose liberal egalitarian values deemed progressive on work relations characterized by deep inequality and dependency. In another contribution to this volume, Bolay and Rey highlight the logistics deployed in the extractive industries to enable the families of expatriate employees to live a cosmopolitan lifestyle while remaining mostly secluded within mining compounds. Such logistics include replicable designs of international schools as well as leisure activities ‘just like at home’, worldwide circulation of a precarious workforce of international schoolteachers, or charities inspired by corporate social responsibility through which expatriates perform their engagement with ‘the locals’.

On the other end of the ladder of privilege, erected walls and security apparatuses cannot cordon off the spatial continuities of an enclave. Outside the realm of privilege, the enclave is discursively constructed through imagined connections to the global world, often accessed using technological devices. In Kagan and Gez’s article, the tablets used by Bridge International Academy in Kenyan townships can be interpreted as an attempt to access the desired ‘international’ arena, whose implications are both practical (expanding a standardized, streamlined approach to teaching) and symbolic (nurturing hopes for international mobility among students and families). Not unlike extractive enclaves, replicability of schools plays a key role in fostering imaginaries of potential circulation between enclaves that run counter to the severe deprivation that dominates the neighbourhoods where these schools are established. Here, it is the aspirational bridging between places rather than boundary work that is at the forefront.

### Enclaves and immunity

By and large, cosmopolitan enclaves are not clearly delineated and monolithic spaces but plural and fragmented ones with shifting boundaries. Also, special (aspirational or concrete) connections tend to be developed between enclaves across the globe. Here, the continuities and overlaps between extractive enclaves as an economic project and cosmopolitan enclaves as a moral project, as suggested by Bolay and Rey in their contribution to this volume, is fruitful. Ferguson (2005: 379) reminds us of the discontinuities of the movement of capital in the extractive sector:

It is worth noting that the movement of capital that is entailed in such enterprises is ‘global’ in the sense that it crosses the globe, but it does not encompass or cover contiguous geographic space. The movements of capital cross national borders, but they jump point to point, and huge areas are simply bypassed.

Rather than a standardization of space that would be the hallmark of states (Scott, 1998), extractive enclaves are one of many manifestations of globalization’s graduated sovereignty. As Appel (2019: 112) argues, extractive enclaves can be understood as a performance of market discipline and standards of efficiency while claiming to avoid entanglements with local life. Enclave boundaries then serve a more general project of dissociating a company’s activities from the expectations and obligations related to the territories where it operates. ‘The separation is a spatial and procedural stage on which companies enact removal from and superiority to the legal, environmental, political, and financial situations in which they are causally and irrevocably implicated’ (Appel, 2019: 114).

This immunity offered by enclaves is certainly a central point of the analysis. It invites us to differentiate between enclaves as a *project* (which is the case for most enclave economies) and enclaves as an *effect of exclusion*. The latter recalls the starting point of Cacciotti’s case study of a public school in Pisacane, a migrant neighbourhood in Rome, which, through xenophobic mediatization and related political controversies, offered an ideal scapegoat for right-wing populist agendas. Unlike the inward strategies associated with the ethnic enclave hypothesis, here families struggled to reverse the stigma of foreignness and turn it into an attractive feature of diversity and cosmopolitanism, both to express ‘openness to the Other’ and out of a ‘desire to belong to a cosmopolitan class’ (Cacciotti, this volume). By actively subverting elitist claims of internationality as a marker of social value and making it their own, the inhabitants of Pisacane progressively attracted cosmopolitan Italians to settle in the neighbourhood and enrol their children in the local school, ironically rebranded ‘international’. Going beyond the opposition between ordinary cosmopolitanism and elites’ cosmopolitanism, Cacciotti’s article demonstrates the ambiguities and malleability of cosmopolitan claims. Though not necessarily carrying the same meaning among the ostracized residents and the newcomers, cosmopolitan aspirations enable a common project that counters xenophobic anxieties about social contamination.

In another context, Bolay and Rey’s contribution invites us to think about the (un)doing of the enclave. The modularity of these spaces, Bolay and Rey show, enables them to pop up and fade away alongside the temporal fixation and redirection of capital on a global scale. For the duration of extractive projects, enclavement is meant to offer a frictionless space for the circulation of people and resources by replicating standardized spaces wherever their locations. The two articles – Cacciotti as well as Bolay and Rey – show how cosmopolitanism is locally grounded and serves as a moral or political project reflecting the interests and worldview of the community that it discursively – and paradoxically – protects from a perceived external threat.

### Enclaves and the domestic side of globalization

As we have seen, cosmopolitan enclaves help to concretize the spatial discontinuities that are necessary for global capitalism to extract and circulate resources or capital. Yet, the concept also allows to address the familial and gender work associated with the male-dominated industries of economic globalization (see Bear et al., 2015). The ideologies of enclavement pervade the space and can also be identified, beyond the sphere of economic activities, in the domestic and the everyday. This domestic space is where postcolonial continuities may seem most obvious. Botelho shows how Scandinavian expats in Maputo fail to abide by the client–patron relationship common in Mozambique that would require them to support their service providers and domestic workers beyond their formal contractual obligations. In this regard, cosmopolitan enclaves significantly differ from immigrant enclaves as theorized by Portes and Manning (1986), where emergency help and informal social mobility ladders, grounded in shared expectations of differentiated obligations, are central to paternalistic work relations.

Education and schools are central in several case studies in this special issue. One of the reasons why this is so lies in the contributors’ choice to distance themselves from normative political projects and instead to analyse the environments in which they are performed. While international schools for the children of expatriate employees (Assa-Inbar; Bolay and Rey) can be approached as an outgrowth of the domestic foundations of global capitalism, self-labelled international schools in Kenyan townships (Kagan and Gez) and in one of Rome’s marginalized neighbourhoods (Cacciotti) address the exclusive use of the category of the ‘international’ by privileged groups. In these cases, underprivileged groups contest their exclusion by discursively appropriating claims of internationalism/cosmopolitanism to access educational, political, and material resources within the reach of their perimeter.

## Enclavement of expatriate communities, strategies of cosmopolitan rebranding and beyond

This special issue is divided into three sections. The first set of articles tackles the *enclavement of expatriate communities* by analysing the underlying architectural, spatial, discursive, interactional, legal, and ethical dimensions of boundary work. These articles analyse how the distinction between ‘locals’ and ‘expats’ translates into symbolic boundaries, which are sometimes reinforced by state regulations, such as illustrated by Chinese international schools (Assa-Inbar). This is further explored by Botelho, who, in her article evokes the tensions created by Scandinavian assumptions of equality when confronted by the moral and social obligations of domestic labour in Maputo, thereby highlighting the ethical dimension of cosmopolitan enclavement. These articles raise questions about the spatiality of expatriate enclaves, as manifesting through geographic urban dimensions or encoded in specific architectures. The second set of articles analyses *strategies of cosmopolitan rebranding* and describes how activists, educational institutions, and business actors all seek to reverse processes of social exclusion, marginalization and ghettoization by rebranding excluded communities using the cosmopolitan idiom. Diversity, multiculturalism, and international labelling thus become specific strategies for pursuing political, economic or social ends. Such contested endeavours have implications regarding how identities are shaped, opening avenues for local pride and hope for betterment. While such endeavours may succeed under some conditions by ‘reversing the stigma’ (Cacciotti), Kagan and Gez demonstrate that such re-labelling may overemphasize an illusionary, aspirational connection to an imagined global world. The third part of the special issue looks *beyond cosmopolitan narratives* and questions the social and economic realities that may either be hidden from, or consolidated by, cosmopolitan discourses. Cosquer reveals the orientalist nature of expatriate representations of otherness inherent in their cosmopolitan desires for difference in Abu Dhabi, and shows how such imaginaries obscure the presence of a silent majority within the country’s resident population, namely non-local temporary South Asian workers. Finally, in the context of the economic activity of multinational companies, Bolay and Rey look beyond the internationalist foundational myth of international schools: after showing their similarities to the extractive industries, they conceptualize international schools as modular enclaves reinforced by cosmopolitan ideals.

To conclude this introduction, we would like to suggest research orientations and analysis that could be further deepened through the concept of cosmopolitan enclaves. First, we would like to invite scholars to explore the various entanglements between cosmopolitan enclaves and states. The idea of states as transcendent political authorities has not disappeared, despite what some observers in the 1990s suggested (Stepputat and Nuijten, 2018). The resurgence of the state as a control and regulation authority has even tended to increase under the recent Covid-19 pandemic. Rather than vanishing, ideas around the transcendent authority of states are transforming (e.g. Hibou, 1999) and we suggest that cosmopolitan enclaves contribute to that transformation, insofar as cosmopolitan enclaves offer spaces in which actors tend to act *as if* they operate outside the nation states. Second, the concept of cosmopolitan enclave can be used to rethink moral, spatial, racial, distinctive and language boundaries, analysing which categories of people, resources, and ideas circulate (or not) both across enclave boundaries and between enclaves. The concept can also be used to analyse discourses, narrations, and practices that circulate across and within enclave boundaries, dominated as they are by sets of dichotomies vis-à-vis the outside world (expat/local; open-minded/narrow-minded). Finally, research along these lines can explore how cosmopolitan enclaves are entangled with the histories of colonialism, race, and gender. In this regard, ethnographic studies may develop a fine-grained analysis of how cosmopolitanism might be cultivated within enclaved spaces, either to secure privilege from outside threats and consolidate power, or – for those who do not benefit from the hyperbolic promises of globalization and are marginalized by the consequences of (post)colonial legacies – to contest power and access opportunities.

## Notes

1. All the authors of this special issue participated in a double panel titled ‘Cosmopolitan enclaves: Tensions and paradoxes,’ which took place in Stockholm in August 2018 at the European Association of Social Anthropologists’ biannual conference.

2. ‘Graduated sovereignties’ refers to ‘differentiated zones unevenly integrated into the structures of state power and global capital’ (Ong, 2006: 91; see also Sidaway, 2007).
